# Mental Health of Refugees and Migrants during the COVID-19 Pandemic: The Role of Experienced Discrimination and Daily Stressors

**DOI:** 10.3390/ijerph18126354

**Published:** 2021-06-11

**Authors:** Eva Spiritus-Beerden, An Verelst, Ines Devlieger, Nina Langer Primdahl, Fábio Botelho Guedes, Antonio Chiarenza, Stephanie De Maesschalck, Natalie Durbeej, Rocío Garrido, Margarida Gaspar de Matos, Elisabeth Ioannidi, Rebecca Murphy, Rachid Oulahal, Fatumo Osman, Beatriz Padilla, Virginia Paloma, Amer Shehadeh, Gesine Sturm, Maria van den Muijsenbergh, Katerina Vasilikou, Charles Watters, Sara Willems, Morten Skovdal, Ilse Derluyn

**Affiliations:** 1 Department of Social Work and Social Pedagogy, Ghent University, 9000 Gent, Belgium; eva.spiritusbeerden@ugent.be (E.S.-B.); An.verelst@ugent.be (A.V.); ines.devlieger@ugent.be (I.D.); 2 Department of Public Health, University of Copenhagen, 1014 Copenhagen, Denmark; nina.primdahl@sund.ku.dk (N.L.P.); m.skovdal@sund.ku.dk (M.S.); 3 Department of Health Education, University of Lisbon, 1400 Lisbon, Portugal; fabioguedes_93@hotmail.com (F.B.G.); margarida.gaspardematos@gmail.com (M.G.d.M.); 4 Department of Biomedical, Metabolical and Neurosciences, University of Modena and Reggio Emilia, 41125 Modena, Italy; antonino.chiarenza@unimore.it; 5 Department of Public Health and Primary Care, Ghent University, 9000 Gent, Belgium; stephanie.demaesschalck@ugent.be (S.D.M.); Sara.Willems@ugent.be (S.W.); 6 Department of Child Health and Parenting, Uppsala University, 75236 Uppsala, Sweden; Natalie.Durbeej@pubcare.uu.se (N.D.); fatumo.osman@pubcare.uu.se (F.O.); 7 Department of Social Psychology, Universidad de Sevilla, 41018 Seville, Spain; rocioga@us.es (R.G.); vpaloma@us.es (V.P.); 8 Institutional Discourse Research Laboratory, National and Kapodistrian University of Athens, 14122 Athens, Greece; ioanel@otenet.gr; 9 Department of Psychology, Maynooth University, W23 F2K8 Co. Kildare, Ireland; rebecca.a.murphy@mu.ie; 10 DIRE Laboratory, Department of Social Sciences, La Reunion University, 97400 Saint-Denis, France; rachid.oulahal@univ-reunion.fr; 11 School of health and Welfare, Dalarna University, 79188 Falun, Sweden; 12 Department of Sociology, University of South Florida, Tampa, FL 33620, USA; padillab@usf.edum; 13 ISCTE-IUL, 1649-026 Lisboa, Portugal; 14 Department of Psychology, Al Istiqlal University, Jericho P 580, Palestine; amershehadeh2008@yahoo.com; 15 Department of Psychology, Université de Toulouse, 31058 Toulouse, France; gesine.sturm@univ-tlse2.fr; 16 Department of Primary and Community Care, Radboud University, 6500 HB Nijmegen, The Netherlands; maria.vandenmuijsenbergh@radboudumc.nl; 17 Research Center for Greek Society, Academy of Athens, 15126 Athens, Greece; k.vassilikou@gmail.com; 18 Department of School of Education and Social Work, University of Sussex, Sussex BN1 4GE, UK; C.Watters@sussex.ac.uk

**Keywords:** refugees, migrants, mental health, discrimination, COVID-19

## Abstract

The COVID-19 pandemic is a defining global health crisis of our time. While the impact of COVID-19, including its mental health impact, is increasingly being documented, there remain important gaps regarding the specific consequences of the pandemic on particular population groups, including refugees and migrants. This study aims to uncover the impact of the COVID-19 pandemic on the mental health of refugees and migrants worldwide, disentangling the possible role of social and daily stressors, i.e., experiences of discrimination and daily living conditions. Descriptive analysis and structural equation modeling were used to analyze the responses of N = 20,742 refugees and migrants on the self-reporting global ApartTogether survey. Survey findings indicated that the mental health of refugees and migrants during the COVID-19 pandemic was significantly impacted, particularly for certain subgroups, (i.e., insecure housing situation and residence status, older respondents, and females) who reported experiencing higher levels of increased discrimination and increases in daily life stressors. There is a need to recognize the detrimental mental health impact of the COVID-19 pandemic on particular refugee and migrant groups and to develop interventions that target their unique needs.

## 1. Introduction

Since the beginning of 2020, the coronavirus disease (COVID-19) has been dominating the world. A year later, more than two million deaths can be attributed to the outbreak [1]. Looking back on previous epidemics and pandemics (e.g., Ebola, Zika, HIV), numerous studies have documented a range of negative mental health outcomes linked to the viruses and diseases, such as experiences of depression, anxiety and post-traumatic stress, affecting both patients and their families [2,3,4]. A study on the mental health impact of the Ebola epidemic on the general population found that increased symptoms of post-traumatic stress disorder (PTSD), depression and anxiety are common, even one year after the outbreak [5]. Moreover, detrimental effects on mental health were not directly related to or limited by the proximity to the infectious outbreak, with studies indicating that elevated fear of contagion related to Ebola were evident in the general public [6,7]. In addition, three years after the SARS-CoV-1 outbreak in China, higher levels of depressive symptoms and alcohol dependence have been reported, especially within the populations that were quarantined or were in high-exposure areas [8].

Unfortunately, COVID-19 seems no different. According to a longitudinal study set in the UK, the mental health of the participants had significantly deteriorated when compared to before the pandemic [9]. In addition, a systematic review of the current existing evidence regarding the mental health impact of COVID-19 found psychiatric symptoms and/or low psychological well-being in patients, health care workers and the general public [10]. While the impact of COVID-19, including its mental health impact, is increasingly being documented [11,12,13], there remain important gaps regarding the specific consequences of the pandemic on particular population groups, such as refugees and migrants. Even in previous epidemics and pandemics, no studies have been carried out that focus on the psychological well-being of refugees and migrants in this context. This is surprising, given the size of this group worldwide: almost 4% of the world population lives outside their country of origin [14] and, according to the United Nations High Commissioner for Refugees (UNHCR), there are 80 million refugees worldwide [15]. Moreover, this lack of knowledge on the mental health impact of epi-/pandemics in this group is also surprising, considering that these populations already suffer from an increased psychological vulnerability [16], and the psychosocial risks presented by a pandemic have potential to further exacerbate their susceptibility to experiencing mental health difficulties.

The social–ecological model of Miller and Rasmussen (2016), a frequently used theoretical model in the field of refugee studies, points to the strong mental health impact of so-called daily stressors that migrants and refugees experience during the migration trajectory and after settlement [17,18]. Research has documented how migrants and refugees are extensively exposed to a cumulative convergence of risk factors which may negatively impact their mental health, such as precarious living situations, living in neighborhoods characterized by social exclusion and material deprivation, experiencing long-lasting uncertainty regarding their legal status and future, and enduring barriers to accessing the labor market and securing economic stability [19,20]. In the context of a pandemic, especially when governments take particular sanitary measures to prevent the further spread of the virus, such as the closure of certain labor market segments (e.g., cafes and restaurants) or schools, the already stressful daily living conditions experienced by socially vulnerable population groups can be further exacerbated. This has already been well documented in the general population in relation to the COVID-pandemic, as rates of poverty are on the rise [21,22].

Further, the social–ecological model of refugees’ and migrants’ distress underscores the importance of assessing environmental stressors that take place in refugees’ and migrants’ post-resettlement lives, specifically indicating stimuli that are harmful and out of a person’s control [18]. They point out to the importance of perceived discrimination, as a harmful recurrent stressor related to the post-migration journey [18,23]. Experiences of discrimination have a well-documented negative effect on victims’ mental health [24,25], such as increased symptoms of anxiety and depression [26]. Moreover, emergencies and their aftermaths often give rise to increasing discrimination [27]. An outbreak of disease, such as COVID-19, generates fear and anxiety, leading to stigma towards particularly affected groups, such as people that have traveled abroad or people of Asian descent [28]. Soon after the coronavirus outbreak, outbursts of racism, xenophobia and stigmatization have been widely reported [29]. Increased discrimination as a result of the COVID-19 pandemic may further contribute to the already deleterious risk of refugees and migrants experiencing mental health difficulties.

This article presents a sub-section of data from the ApartTogther study, which aimed to assess the mental health of refugees and migrants in the context of a pandemic, hereby looking at particular aspects of their lives that already have been documented to influence the mental health of these groups, such as increased discrimination and additional daily stressors. Through exploring the impact of COVID-19 on the mental health of refugees and migrants, we aim to identify who within these populations is most at risk and why. It is argued that the study findings could consequently inform the strategic selection of targeted and context-responsive psycho-social interventions for the refugees and migrants that are most at risk.

## 2. Materials and Methods

### 2.1. Study Design

A quantitative global study was performed to assess the mental health of refugees and migrants during the COVID-19 pandemic. Data was collected in an online global survey, as part of the ApartTogether study, a collaboration between a large European consortium of academics and, as of June 2020, the World Health Organization (WHO) [30]. An iterative back-translation was carried out in these 37 languages, namely English (base language), Amharic, Arabic, Bengali, Bosnian, Bulgarian, Chinese, Croatian, Danish, Dutch, French, German, Hindi, Italian, Kurdish (Kurmanji), Oromo, Pashto, Persian, Polish, Portuguese, Portuguese (Brazilian), Romanian, Russian, Serbian, Spanish, Swahili, Swedish, Tagalog, Tamil, Tigrinya, Turkish, Ukrainian, Urdu, and Yoruba. The online survey ran from April 2020 until November 2020. Ethics approval for the entire project was granted by the Ethics Committee of the Faculty of Psychology and Educational Sciences of Ghent University and by the WHO Ethics Review Committee.

### 2.2. Study Population and Procedure

In total, N = 20,742 participants entered the survey; however, given the participants could stop the survey at any time, not everyone completed all items. All participants were older than 16 years old. Table 1 shows the socio-demographic characteristics of the participants in this study. ‘Citizen science’, understood as the public’s participation in scientific research, was a primary recruitment strategy [31]. An extensive media campaign was rolled out to reach participants through Facebook, Twitter and WhatsApp and networks of researchers and individuals associated with the target population were informed to encourage participation. In addition, in countries with a large number of refugees, local enumerators were contracted to further the dissemination of the survey, particularly within harder-to-reach groups. Participation in the survey was entirely voluntary. Informed consent, indicating the aims and conditions of the survey, needed to be approved at the beginning of the survey. At the end of the survey, people were given contact details of the research team, as well as a link to the WHO-website to have more information on COVID-19.

### 2.3. Study Questionnaire

The current study focuses on four different parts of the ApartTogether survey, socio-demographic characteristics, mental health, daily stressors and social well-being.

Participants completed questions about sociodemographic characteristics, including age, gender, housing situation (i.e., living in a house or apartment, asylum center, refugee camp or on the street/in an insecure accommodation), and residence status (i.e., citizen, permanent documents, temporary documents, no documents).

Further, respondents were asked to indicate whether their mental health has deteriorated, remained the same or improved on a scale from 0 to 2 for 11 mental-health-related items. These items inquired about feelings of depression, anxiety, worries, feelings of loneliness, anger, unpleasant reminders of past traumatic experiences, physical reactions to stress, feelings of irritation, hopelessness, sleeping problems, and substance use, such as alcohol and drugs.

Third, to measure the experiences of discrimination of respondents, six items were created: being differently treated because of your origin, being called names because of your origin or religion, being avoided, other people being anxious of you, being unfairly treated by the police, and being treated with kindness. Respondents were asked to indicate, on a scale of 0 to 2, whether they felt their treatment by others had gotten worse, stayed the same or had improved since the outbreak of the coronavirus. Finally, respondents were asked to report whether their daily living conditions had deteriorated, remained the same, or improved on a scale from 0 to 2. Items regarding daily stressors consisted of housing situation, access to work, feelings of safety, food, clothes, financial means, medical care, health situation, support from non-governmental organizations (NGO’s) and other organizations, relationship with partner, and relationship with children.

### 2.4. Statistical Analysis

Descriptive statistics were used to describe participants’ socio-demographics in relation to the considered variables.

Previous to the structural equation modeling, an exploratory factor analysis (EFA) was conducted on the different items created in the questionnaire to test the factor structure for the included dimensions. Next, a confirmatory factor analysis (CFA) was performed to determine if the theorized measurement model shows an acceptable fit for each latent construct. To check for common method bias in the self-reported questionnaire, two separate tests were conducted. First, Harman’s single factor test was performed, with a variance of less than 50%, indicating that a common method bias should not be a problem [32]. In addition, the common latent factor technique was used as a second check, with a variance of less than 50% again indicating no problem of common method bias [32].

The package used for the EFA and CFA was the lavaan package, version 0.6–8 [33]. The weighted least squares mean- and variance-adjusted (WLSMV) estimator was used, implying diagonally weighted least squares (DWLS) to estimate model parameters and the full weight matrix was used to compute robust standard errors and a mean- and variance-adjusted test statistics. The following fit indices were used: the chi-square test statistic and p-value, Root Means Square Error of Approximation (RMSEA), Standardized Root Means Square Residual (SRMR), Tucker–Lewis Index (TLI), and Comparative Fit Index (CFI). For the RMSEA, a value below 0.06 is required for a good fit [34] and a value below 0.08 indicates an acceptable fit [35]. For the SRMR, a value below 0.08 was recommended [34]. For CFI and TLI, values above 0.95 indicate a good fit, while values above 0.90 indicate an acceptable fit to the data [34,36,37]. If these fit indices were not adequate, the measurement models were adjusted based on the standardized factor loadings or on the modification indices. Cronbach’s alpha was used to test the reliability of the scales, a value of 0.70 or higher indicating a satisfactory level of reliability [38].

Finally, a structural equation model (SEM) was used to examine the full model fit, again using the lavaan package version 0.6–8 [33] and the ‘WLSMV’-estimators. Further, the structural equation model (SEM) was used to examine the predictors of mental health, including the relation between mental health and experienced discrimination, daily stressors and sociodemographic characteristics (i.e., age, gender, housing situation, and residence status) in the context of a pandemic.

To deal with missing data, multiple imputation was performed, using the R-package mice, version 3.12 [39]. Rubin’s (1987) rules were used to pool point estimates and standard error estimates across five imputed datasets [40]. The fit of the model to the data was evaluated, using the same fit indices as previously mentioned.

## 3. Results

### 3.1. Descriptive Statistics

Some descriptive analyses, using crosstabulation, were conducted to get an overview of the differences between the different housing situations and residence statuses in reported deterioration of mental health, experienced discrimination, and experienced daily stressors. In Table 2, an overview for residence status can be found. It is notable that a higher percentage of respondents within the more insecure residence statuses tend to report a deterioration of their condition on all three topics. The same tendencies are found within the different housing situations (see Table 3), where a higher percentage of respondents that are living in more precarious circumstances (i.e., asylum centers, on the street or in insecure accommodations) report a deterioration in their experiences regarding mental health, daily stressors, and discrimination since the pandemic began.

### 3.2. Exploratory Factor Analysis

Since no standardized questionnaire was used, an EFA was conducted to uncover new underlying constructs from the measured items in the questionnaire. For the EFA-procedure, a principal component analysis was used to determine the number of factors by merging items that have relatively high factor loads. The eigenvalues and factor loadings can be found in Table 4 and Table 5. For the mental health items, the principal component analysis with varimax rotation suggested a two-factor solution, with feelings of depression, anxiety, worry, and loneliness for factor 1, and anger, unpleasant reminders of past experiences, physical reactions to stress, being irritable, feeling hopeless, sleep problems, and substance use for factor 2. The TLI = 0.978 and RMSEA = 0.063 are good, which means two factors are sufficient. The EFA on the six discrimination items suggested a one-factor solution, TLI = 0.91 and RMSEA = 0.15. The RMSEA should be below 0.08; however, this does not improve with two factors. For the different items regarding the daily stressors of refugees and migrants, the EFA suggested a three-factor solution, with factor 1 consisting of housing, work, safety, food, clothes and financial means, factor 2 consisting of NGO support, relationship with partner and relationship with children, and factor 3 of access to medical care and health situation. For item 7 (i.e., NGO support) and item 9 (i.e., health situation) it is less clear to which factor they belong but given the context it was decided to place item 7 within factor 2 and item 9 withing factor 3. The EFA with three factors for the daily stressor items resulted in TLI = 0.915 and RMSEA = 0.10. Again, the RMSEA index is too high, but using four factors makes it worse.

### 3.3. Confirmatory Factor Analysis

Following the EFA procedure, a CFA was conducted to confirm the EFA. Details on the fit indices of the model can be found in Table 6.

#### 3.3.1. Mental Health

This two-factor model (i.e., factor 1 = symptoms of anxiety and depression and factor 2 = symptoms of hyper arousal) of mental health showed a good model fit, TLI = 0.99, CFI = 0.99, RMSEA = 0.04, SRMR = 0.02. Cronbach’s α for factor 1 is 0.94 and for factor 2 is 0.95, indicating sufficient reliability of the scales.

#### 3.3.2. Perceived Discrimination

This one-factor model with six items showed a moderate model fit. Further inspection of the modification indices suggesting allowing a correlation between items 4 and 5 (i.e., respectively ‘others avoid me’ and ‘others seem to be anxious about me’). The model showed good fit, TLI = 0.99, CFI = 0.99, RMSEA = 0.07, SRMR = 0.02. Cronbach’s α for the discrimination scale is 0.93, indicating sufficient reliability.

#### 3.3.3. Daily Stressors

The three-factor model of daily stressors showed a good fit at first inspection; however, SRMR = 0.055 is a little too high. Further inspection of the modification indices suggests allowing a correlation between items 10 and 11 (i.e., respectively ‘relationship with partner’ and ‘relationship with children’). This model showed a good fit, TLI = 0.99, CFI = 0.99, RMSEA = 0.05, SRMR = 0.03. The reliability test showed a Cronbach’s α of 0.92 for factor 1, α = 0.84 for factor 2, and α = 0.85 for factor 3, again indicating sufficient reliability for these scales.

#### 3.3.4. Common Method Bias

The proportion of the variance explained by the factor in the Harman’s single factor test is 44%, indicating that the common method bias should not be a problem. A second check was performed using the common latent factor technique, which showed a common variance of 1%, indicating that the common method bias is not a problem. Therefore, it is possible to proceed with the analysis using the factors mentioned above.

#### 3.3.5. Full SEM Model

Lastly, a structural equation model (SEM) is used to examine the fit of the proposed models and to inspect the predictors of mental health. The structural equation model that was used showed a good fit to the data, TLI = 0.99, CFI = 0.99, RMSEA = 0.02, SRMR = 0.04.

### 3.4. Differences in Mental Health Outcomes

In Table 7 the regressions of the SEM analysis can be found. Since response values for the mental health items were 0 = worse than before, 1 = same as before, and 2 = better than before, scoring higher on the two mental health scales should be interpreted as showing less symptoms of deteriorated mental health.

The same significance and effects were found in both mental health scales, meaning that the considered variables had the same impact on both anxiety and depression, and on hyper-arousal. First, age showed a significant mediating effect on mental health outcomes. Older participants of the survey reported a significantly lower score, indicating that younger participants report a less negative effect of COVID-19 on their anxiety and depression, and on hyper-arousal. Next, a significant effect between gender and mental health outcome was demonstrated, with males reporting significantly fewer negative impacts of COVID-19 on their mental health outcomes, as compared to females. As regards the relationship between housing situations and mental health outcomes, again a significant effect was found. Specifically, those living in asylum centers and respondents living on the street or in insecure accommodation reported the worst effect of COVID-19 on their mental health. No significant difference was found between living in an asylum center and living on the street or in insecure accommodation. Respondents in refugee camps reported the least effect of COVID-19 on their feelings of anxiety and depression. Further, it was found that residence status had a significant impact on the reported mental health outcomes. Respondents with citizenship scored highest on both the anxiety and depression scale, and on the hyper-arousal scale, meaning that they reported the least effect of COVID-19 on their mental health.

Looking at the impact of experienced discrimination on respondents’ anxiety and depression, and hyper-arousal, a significant effect was also found. Specifically, respondents who perceived that discrimination towards them had become worse since the pandemic, also reported their mental health to have become worse.

Lastly, all three of the different daily stressor scales indicated a significant effect on the reported mental health outcomes. Respondents who felt that their stress concerning basic needs had become worse since the pandemic also reported a deterioration of mental health. Similar results are found for respondents that reported more stress concerning their medical needs. Surprisingly, the results showed that respondents reporting less stress concerning their social needs (i.e., NGO support, relationship with children, and relationship with partner) reported worse feelings of anxiety and depression.

## 4. Discussion

This study is the first to assess refugees’ and migrants’ mental health in the context of the COVID-19 pandemic, including the role of experienced discrimination and daily stressors. Perceived discrimination was found to have a significant effect on both the anxiety and depression scale, and on the hyper-arousal scale of mental health. Respondents that perceived discrimination to have become worse since the pandemic also report worse mental health outcomes. This is consistent with existing research where exposure to discrimination has been linked to higher levels of mental distress [24,25]. Specifically, in the context of a pandemic where minority groups endure discrimination with elevated frequency [29,41].

When it comes to the experiences of daily stressors, all three scales of daily stressors showed a significant impact on mental health. First, regarding basic needs, such as housing, work, food and clothes, this study found that participants reporting the most challenges with regard to securing these basic needs also reported a deterioration in their feelings of anxiety and depression, and their feelings of hyper-arousal. Likewise, respondents that reported more difficulties with their medical needs during the COVID-19 pandemic, reported suffering more from negative mental health outcomes on both mental health scales. These findings support a wide array of previous studies that found material and situational daily stressors significantly impacting people’s mental health—see, e.g., [17,42]. Further, results of the survey analysis showed that respondents whose social needs were better fulfilled, reported being worse on both mental health scales. Possible explanations for this are threefold. Social support has been found to be extremely important when coping with mental health distress by a great body of research [43,44]. Migrants, who often face high barriers to access formal support, tend to draw upon informal networks for support in times of high mental health distress [45]. Alternatively, previous research has highlighted the complexity of social support in situations with high levels of daily challenges [46]. It states that avoidant coping strategies in highly stressful situations might be the most helpful to overcome psychological distress. Lastly, the “pressure cooker effect” could be at play [47,48]. Research found that social support, specifically talking about trauma and distress with people in the same situation, might exacerbate distress.

The sociodemographic characteristics included in this study all showed a significant impact on both mental health scales. In the current study, younger respondents reported a less negative effect on their mental health, which supports previous findings. For example, older age has been linked to decreased resilience and lower levels of mental health in displaced populations [49]. However, it is important to note that this study did not include children and only a small group of young adolescents, which is a population of refugees and migrants that have been found to be particularly vulnerable for mental health in research prior to the pandemic—see, e.g., [50,51]. In addition, this study indicated that COVID-19 had a more deleterious effect on women’s mental health outcomes as compared to males. According to previous studies, refugee and migrant women, especially in low-income countries, were already at risk for negative mental health outcomes [19,52,53]. On top of that, gender differences in health risks are exacerbated during the pandemic with certain negative health outcomes particularly impacting women (e.g., gender-based violence, drug-development, mental health) [10,54]. Further, the current study identified the most at-risk cohorts within refugee and migrant populations. Respondents that have no documents or temporary documents, and respondents that live on the street, in insecure accommodation, or in asylum centers, reported comparatively worse mental health outcomes as a result of the COVID-19 pandemic. In studies conducted before the pandemic, it was already apparent that sub-cohorts of refugees and migrants living in comparatively more precarious situations are at higher risk to develop negative mental health outcomes [55].

### Strengths, Limitations and Future Directions

This research forms an important steppingstone in highlighting the mental health conditions of refugees and migrants during the COVID-19 pandemic, and to uncover important factors that play a significant role in shaping the psychological experiences of this population. Given the sample size and the global outreach of the survey, the current study is an important addition to the academic work on refugees’ and migrants’ mental health. Moreover, the exceptional conditions in which this survey took place help to shape necessary policy recommendations to move forward.

There are several limitations to this study that need to be mentioned. A first shortcoming of the current study lies within the method of recruitment. Due to the method of recruitment, which primarily took place online, via social media, and the COVID-19 restrictive measures, it was easier to reach a younger population with higher literacy and access to technical devices. It was more difficult to contact populations of harder-to-reach groups, which are possibly the most impacted by the pandemic. Additionally, to reach refugees and migrants across the globe it was essential to disseminate the survey in a wide range of languages. Whilst the survey was translated and disseminated in 37 languages, additional translations would have certainly facilitated reaching even more people. Further, the survey was active over a seven-month period, meaning that respondents entered at different epidemiological stages, which varied both between countries and over time. Last, in order to frame the migration situation of the respondents, the survey chose to differentiate between different levels of housing situation and residence status. The survey does not conceptualize the difference between refugees and migrants, making it impossible to compare both groups.

The findings of the current study are important for both research and practice, as they shed a light on some vital aspects impacting refugees’ and migrants’ mental health in the context of a global health pandemic. Future research should further study the interrelations between these different risk factors and mental health, as well remaining aware of the contextual elements that shape the pandemic. For example, we could see whether other models on the impact of stressors on individuals’ mental health and coping strategies, such as the Lazarus and Folkman’s stress and coping model (1984), can be validated in the context of global impact events, such as a pandemic. In addition, it would be interesting to include possible protective factors for refugees’ and migrants’ mental health in the future. Again, theoretical models, such as the Conservation of Resources Theory, could be inspiring or could even be validated in the wake of such a particular event. Including qualitative data in future mental health research with refugees and migrants will support the quantitative findings and help build a narrative that remains true to refugees’ and migrants’ voices. This would be an important aspect of finding ways to cultivate refugees’ and migrants’ mental health when faced with accumulating stressors. Including qualitative data in future mental health research with refugees and migrants in the context of a global health pandemic will further elaborate the quantitative findings and help build a narrative that is attuned to the lived realities and experiences of refugees’ and migrants’. Going forward, it is important to include contextual factors in both research and practice, that might impact mental health.

Given that a higher risk of experiencing negative mental health outcomes was reported by refugees and migrants living in comparatively more precarious situations, the study findings clearly indicate that the COVID-19 pandemic disproportionally impacts the most socially vulnerable sections of society. These reported mental health rates call for targeted interventions, specifically targeting more vulnerable groups (i.e., those living in asylum centers or on the street, those without documents or with temporary documents). First, the living conditions of these high-risk groups, especially undocumented migrants and those living on the street and in unsecured housing situations need to be improved, in terms of their access to proper housing, clothing and food, their work situation (both in terms of access to proper jobs and overall working conditions), and their access to health care. Second, for these groups, effective political action needs to be taken to ensure access to mental health care services. A possibility could be to start with outreaching psychological support in informal camps of transit migrants or in shelters for homeless people. Additionally, the increased mental health vulnerability of refugees and migrants living in asylum centers and refugee camps necessitate a broader availability of psychosocial interventions in these settings. Third, it is important to be more mindful of the detrimental effect of discrimination on mental health. Interventions that aim to mitigate the mental health impact of the COVID-19 pandemic for refugees and migrants should include actions that reduce discrimination. 

## 5. Conclusions

To conclude, this study contributes to the understanding of refugees’ and migrants’ mental health in the context of a pandemic, where refugees and migrants who suffer from increased discrimination and elevated stress regarding material and medical daily needs report an elevated deterioration of their mental health during the COVID-19 pandemic. In addition, refugees and migrants with a more insecure housing situation and residence status are particularly susceptible to experiencing mental health distress.

## Figures and Tables

**Table 1:** Demographic characteristics of the participants.

Variables	N	%
**Gender**		
Male	11,946	57.6
Female	8696	41.9
**Residence status**		
Citizen	5905	28.5
Permanent documents	5294	25.5
Temporary documents	7702	37.1
No documents	1472	7.1
**Housing situation**		
House or apartment	18,260	88.0
Asylum center	430	2.1
Refugee camp	1428	6.9
On the street—insecure accommodation	230	1.1

**Table 2:** Percentage of respondent reporting a deterioration in their condition in relation to residence status.

	Citizen	Permanent Documents	Temporary Documents	No Documents—Undocumented
**Mental health**				
Depressed	50.9%	60.2%	61.9%	59.1%
Worry	52.1%	61.1%	59.7%	61.1%
Anxiety	49.2%	57.0%	59.2%	58.6%
Loneliness	43.7%	54.0%	56.1%	55.1%
Anger	37.8%	47.6%	49.3%	49.5%
Reminders	31.7%	42.2%	44.9%	47.7%
Physical stress reactions	31.6%	41.4%	43.4%	45.6%
Irritable	38.5%	45.6%	46.8%	46.4%
Hopelessness	38.2%	46.1%	49.1%	52.0%
Sleep problems	35.3%	40.6%	47.2%	48.4%
Drugs and alcohol	16.4%	26.8%	25.2%	34.3%
**Daily stressors**				
Housing	27.0%	24.4%	32.1%	47.9%
Work	45.6%	48.2%	53.9%	57.7%
Safety	57.1%	57.9%	56.6%	60.8%
Food	28.3%	25.3%	33.4%	43.2%
Clothes	22.8%	22.5%	24.4%	39.9%
Financial means	54.1%	47.8%	55.2%	60.0%
NGO + other support	25.3%	24.4%	25.1%	35.6%
Medical care	30.6%	25.8%	33.3%	42.4%
Health situation	25.8%	27.1%	31.6%	34.2%
Relationship with partner	14.4%	20.0%	16.2%	20.8%
Relationship with children	10.2%	18.0%	13.1%	16.7%
**Perceived discrimination**				
Treated differently because of origin	10.1%	14.4%	16.4%	24.9%
Treated with kindness	6.7%	11.6%	11.3%	15.0%
Called names because of origin/religion	4.6%	10.4%	11.0%	16.2%
Being avoided	9.4%	15.4%	18.2%	21.7%
Being anxious about me	9.1%	13.1%	15.9%	19.4%
Unfair treatment police	4.2%	9.0%	7.1%	15.6%

**Table 3:** Percentage of respondents reporting a deterioration in their condition in relation to housing situation.

	House or Apartment	Asylum Center	Refugee Camp	On the Streets—Insecure
**Mental health**				
Depressed	59.1%	69.6%	43.4%	65.3%
Worry	58.6%	71.5%	46.2%	64.3%
Anxiety	56.4%	68.3%	43.5%	64.2%
Loneliness	52.5%	65.9%	41.0%	60.8%
Anger	45.2%	64.3%	42.5%	60.9%
Reminders	40.8%	57.7%	33.6%	54.1%
Physical stress reactions	39.5%	55.3%	33.9%	59.3%
Irritable	43.7%	58.5%	42.9%	52.0%
Hopelessness	45.4%	60.1%	40.6%	58.1%
Sleep problems	42.0%	54.2%	41.9%	51.9%
Drugs and alcohol	22.8%	33.2%	24.2%	46.8%
**Daily stressors**				
Housing	29.8%	49.6%	31.0%	56.0%
Work	50.1%	66.8%	45.4%	68.9%
Safety	58.3%	61.9%	44.5%	67.7%
Food	29.1%	40.6%	40.5%	60.2%
Clothes	23.5%	42.4%	27.7%	52.6%
Financial means	53.5%	54.1%	48.5%	73.8%
NGO + other support	25.2%	36.8%	27.1%	46.8%
Medical care	30.8%	47.4%	31.0%	56.7%
Health situation	28.9%	35.8%	24.6%	48.6%
Relationship with partner	16.6%	30.4%	20.0%	44.2%
Relationship with children	12.7%	25.1%	18.3%	34.0%
**Perceived discrimination**				
Treated differently because of origin	13.7%	26.6%	19.8%	31.5%
Treated with kindness	9.7%	14.8%	12.7%	21.1%
Called names because of origin/religion	8.8%	16.4%	12.8%	18.8%
Being avoided	14.1%	24.6%	21.4%	23.7%
Being anxious about me	12.7%	23.7%	15.5%	22.7%
Unfair treatment police	6.5%	12.6%	13.8%	21.1%

**Table 4:** Exploratory factor analysis.

	Eigenvalue	Proportion	Cumulative
**Mental health**			
Factor 1—Anxiety and depression	4.31	0.39	0.55
Factor 2—Hyper-arousal	3.54	0.32	1.00
**Discrimination**			
Factor 1—Discrimination	3.91	0.65	
**Daily stressors**			
Factor 1—Basic needs	3.53	0.32	0.48
Factor 2—Social needs	2.01	0.18	0.76
Factor 3—Medical needs	1.80	0.16	1.00

**Table 5:** Factor loadings, varimax rotated.

Variable	Item	Factor 1	Factor 2	Factor 3	Uniqueness
**Mental health**					
	Depression	0.72			0.31
	Worry	0.78			0.22
	Anxiety	0.75			0.22
	Loneliness	0.61			0.31
	Anger		0.65		0.29
	Reminders		0.64		0.34
	Physical stress reactions		0.74		0.24
	Irritable		0.75		0.23
	Hopelessness		0.71		0.28
	Sleep problems		0.71		0.33
	Substance use		0.70		0.38
**Discrimination**					
	Treated differently	0.82			0.33
	Treated with kindness	0.76			0.43
	Called names	0.85			0.28
	Avoided	0.82			0.32
	Others are anxious	0.84			0.29
	Police treatment	0.75			0.44
**Daily stressors**					
	Housing	0.63			0.50
	Work	0.74			0.40
	Safety	0.66			0.49
	Food	0.77			0.31
	Clothes	0.70			0.39
	Financial means	0.72			0.44
	NGO support		0.40	0.44	0.53
	Medical access			1.05	−0.22
	Health situation	0.51		0.47	0.46
	Relationship partner		0.84		0.22
	Relationship children		0.90		0.12

**Table 6:** Fit indices for the CFA.

	TLI	CFI	RSMEA	SRMR
Mental health	0.99	0.99	0.04	0.02
Discrimination	0.99	0.99	0.07	0.02
Daily stressors	0.99	0.99	0.05	0.03
Full SEM model	0.99	0.99	0.02	0.04

**Table 7:** Structural equation modeling—regressions.

	ß	SE	t	df	*p*
**Mental health—anxiety and depression**					
Age	−0.003	0.001	−3.487	1508.506	0.001 **
Male	0.110	0.018	6.142	79.775	0.000 ***
House or apartment	0.238	0.063	3.781	993.853	0.000 ***
Refugee camp	0.523	0.070	7.500	309.989	0.000 ***
On the street—insecure accommodation	0.167	0.099	1.677	259.769	0.095
Permanent documents	−0.094	0.024	−3.978	104.899	0.000 ***
Temporary documents	−0.205	0.022	−9.239	399.862	0.000 ***
No documents	−0.119	0.037	−3.192	35.623	0.003 **
Discrimination	0.336	0.027	12.418	9.297	0.000 ***
Daily stressors—basic needs	0.520	0.023	23.020	1867.066	0.000 ***
Daily stressors—social needs	−0.411	0.069	−5.919	9.840	0.000 ***
Daily stressors—medical needs	0.255	0.045	5.614	15.463	0.000 ***
**Mental health—hyper-arousal**					
Age	−0.002	0.001	−2.647	487.040	0.008 **
Male	0.068	0.017	3.909	135.512	0.000 ***
House or apartment	0.281	0.058	4.831	1352.808	0.000 ***
Refugee camp	0.428	0.065	6.630	1045.357	0.000 ***
On the street—insecure accommodation	0.131	0.095	1.386	218.742	0.167
Permanent documents	−0.088	0.023	−3.792	58.559	0.000 ***
Temporary documents	−0.198	0.022	−9.193	118.909	0.000 ***
No documents	−0.167	0.036	−4.616	188.655	0.000 ***
Discrimination	0.410	0.023	17.835	44.130	0.000 **
Daily stressors—basic needs	0.369	0.021	17.941	20.073	0.000 ***
Daily stressors—social needs	−0.203	0.058	−3.516	95.743	0.001 **
Daily stressors—medical needs	0.160	0.039	4.108	676.817	0.000 ***

** *p* < 0.01; *** *p* < 0.001.

## Data Availability

Data are not available due to sensitive and personal data.

## References

[1] World Health Organization (2021). WHO COVID-19 Dashboard. https://covid19.who.int/.

[2] Tucci V., Moukaddam N., Meadows J., Shah S., Galwankar S.C., Kapur G.B. (2017). The forgotten plague: Psychiatric manifestations of ebola, zika, and emerging infectious diseases. J. Glob. Infect. Dis..

[3] Remien R.H., Stirratt M.J., Nguyen N., Robbins R.N., Pala A.N., Mellins C.A. (2019). Mental health and HIV/AIDS. AIDS.

[4] Kamara S., Walder A., Duncan J., Kabbedijk A., Hughes P., Muana A. (2017). Mental health care during the Ebola virus disease outbreak in Sierra Leone. Bull. World Health Organ..

[5] Jalloh M.F., Li W., Bunnell R.E., Ethier K.A., O’Leary A., Hageman K.M., Sengeh P., Jalloh M.B., Morgan O., Hersey S. (2018). Impact of Ebola experiences and risk perceptions on mental health in Sierra Leone, July 2015. BMJ Glob. Health.

[6] Esterwood E., Saeed S.A. (2020). Past Epidemics, Natural Disasters, COVID19, and Mental Health: Learning from History as we Deal with the Present and Prepare for the Future. Psychiatr. Q..

[7] Morganstein J.C., Ursano R.J. (2020). Ecological Disasters and Mental Health: Causes, Consequences, and Interventions. Front. Psychiatry.

[8] Liu X., Kakade M., Fuller C.J., Fan B., Fang Y., Kong J., Guan Z., Wu P. (2012). Depression after exposure to stressful events: Lessons learned from the severe acute respiratory syndrome epidemic. Compr. Psychiatry.

[9] Pierce M., Hope H., Ford T., Hatch S., Hotopf M., John A., Kontopantelis E., Webb R., Wessely S., McManus S. (2020). Mental health before and during the COVID-19 pandemic: A longitudinal probability sample survey of the UK population. Lancet Psychiatry.

[10] Vindegaard N., Benros M.E. (2020). COVID-19 pandemic and mental health consequences: Systematic review of the current evidence. Brain Behav. Immun..

[11] Kumar A., Nayar K.R. (2021). COVID 19 and its mental health consequences. J. Ment. Health.

[12] Khan K.S., Mamun M.A., Griffiths M.D., Ullah I. (2020). The Mental Health Impact of the COVID-19 Pandemic Across Different Cohorts. Int. J. Ment. Health Addict..

[13] Xiong J., Lipsitz O., Nasri F., Lui L.M.W., Gill H., Phan L., Chen-Li D., Iacobucci M., Ho R., Majeed A. (2020). Impact of COVID-19 pandemic on mental health in the general population: A systematic review. J. Affect. Disord..

[14] United Nations International Migration 2020 Highlights. https://www.un.org/en/desa/international-migration-2020-highlights#:~:text=Growth%20in%20the%20number%20of.

[15] UNHCR UNHCR—Refugee Statistics. https://www.unhcr.org/refugee-statistics/.

[16] Hameed S., Sadiq A., Din A.U. (2018). The Increased Vulnerability of Refugee Population to Mental Health Disorders. Kans. J. Med..

[17] Miller K.E., Rasmussen A. (2010). War exposure, daily stressors, and mental health in conflict and post-conflict settings: Bridging the divide between trauma-focused and psychosocial frameworks. Soc. Sci. Med..

[18] Miller K.E., Rasmussen A. (2017). The mental health of civilians displaced by armed conflict: An ecological model of refugee distress. Epidemiol. Psychiatr. Sci..

[19] Porter M., Haslam N. (2005). Predisplacement and Postdisplacement Factors Associated with Mental Health of Refugees and Internally Displaced Persons. JAMA.

[20] Walther L., Fuchs L.M., Schupp J., Von Scheve C. (2020). Living Conditions and the Mental Health and Well-being of Refugees: Evidence from a Large-Scale German Survey. J. Immigr. Minor. Health.

[21] Pereira M., Oliveira A.M. (2020). Poverty and food insecurity may increase as the threat of COVID-19 spreads. Public Health Nutr..

[22] Whitehead M., Taylor-Robinson D., Barr B. (2021). Poverty, health, and covid-19. BMJ.

[23] Ellis B.H., Macdonald H.Z., Lincoln A.K., Cabral H.J. (2008). Mental health of Somali adolescent refugees: The role of trauma, stress, and perceived discrimination. J. Consult. Clin. Psychol..

[24] Pascoe E.A., Richman L.S. (2009). Perceived discrimination and health: A meta-analytic review. Psychol. Bull..

[25] Schmitt M.T., Branscombe N., Postmes T., Garcia A. (2014). The consequences of perceived discrimination for psychological well-being: A meta-analytic review. Psychol. Bull..

[26] Baranik L.E., Hurst C.S., Eby L.T. (2018). The stigma of being a refugee: A mixed-method study of refugees’ experiences of vocational stress. J. Vocat. Behav..

[27] Guadagno L. (2020). Migrants and the COVID-19 Pandemic: An Initial Analysis. https://publications.iom.int/system/files/pdf/mrs-60.pdf.

[28] Turner-Musa J., Ajayi O., Kemp L. (2020). Examining Social Determinants of Health, Stigma, and COVID-19 Disparities. Healthcare.

[29] Dubey S., Biswas P., Ghosh R., Chatterjee S., Dubey M.J., Chatterjee S., Lahiri D., Lavie C.J. (2020). Psychosocial impact of COVID-19. Diabetes Metab. Syndr. Clin. Res. Rev..

[30] World Health Organization (2020). ApartTogether Survey Preliminary Overview of Refugees and Migrants Self-Reported Impact of COVID-19. https://apps.who.int/iris/bitstream/handle/10665/337931/9789240017924-eng.pdf?sequence=1&isAllowed=y.

[31] Irwin A. (1995). Citizen Science: A Study of People, Expertise and Sustainable Development.

[32] Eichhorn B.R. (2014). Common Method Variance Techniques.

[33] Rosseel Y. (2012). Lavaan: An R Package for Structural Equation Modeling. J. Stat. Softw..

[34] Hu L.T., Bentler P.M. (1999). Cutoff criteria for fit indexes in covariance structure analysis: Conventional criteria versus new alternatives. Struct. Equ. Model. Multidiscip. J..

[35] Schreiber J., Nora A., Stage F.K., Barlow E.A., King J. (2006). Reporting Structural Equation Modeling and Confirmatory Factor Analysis Results: A Review. J. Educ. Res..

[36] Brown T.A. (2015). Confirmatory Factor Analysis for Applied Research.

[37] Browne M.W., Cudeck R. (1992). Alternative ways of assessing model fit. Sociol. Methods Res..

[38] Heo M., Kim N., Faith M.S. (2015). Statistical power as a function of Cronbach alpha of instrument questionnaire items. BMC Med. Res. Methodol..

[39] van Buuren S., Groothuis-Oudshoorn K. (2011). Mice: Multivariate Imputation by Chained Equations in R. J. Stat. Softw..

[40] Rubin D.B. (1988). An Overview of Multiple Imputation. http://citeseerx.ist.psu.edu/viewdoc/download?doi=10.1.1.565.6832&rep=rep1&type=pdf.

[41] Demirtaş-Madran H.A. (2020). Exploring the Motivation Behind Discrimination and Stigmatization Related to COVID-19: A Social Psychological Discussion Based on the Main Theoretical Explanations. Front. Psychol..

[42] Ponnamperuma T., Nicolson N.A. (2018). The Relative Impact of Traumatic Experiences and Daily Stressors on Mental Health Outcomes in Sri Lankan Adolescents. J. Trauma. Stress.

[43] Renner A., Hoffmann R., Nagl M., Roehr S., Jung F., Grochtdreis T., König H.-H., Riedel-Heller S., Kersting A. (2020). Syrian refugees in Germany: Perspectives on mental health and coping strategies. J. Psychosom. Res..

[44] Schweitzer R., Melville F., Steel Z., Lacherez P. (2006). Trauma, Post-Migration Living Difficulties, and Social Support as Predictors of Psychological Adjustment in Resettled Sudanese Refugees. Aust. N. Z. J. Psychiatry.

[45] Donnelly T.T., Hwang J.J., Este D., Ewashen C., Adair C., Clinton M. (2011). If I Was Going to Kill Myself, I Wouldn’t Be Calling You. I am Asking for Help: Challenges Influencing Immigrant and Refugee Women’s Mental Health. Issues Ment. Health Nurs..

[46] Verelst A., Bal S., De Schryver M., Kana N.S., Broekaert E., Derluyn I. (2020). The Impact of Avoidant/Disengagement Coping and Social Support on the Mental Health of Adolescent Victims of Sexual Violence in Eastern Congo. Front. Psychiatry.

[47] Hobfoll S.E., London P. (1986). The Relationship of Self-Concept and Social Support to Emotional Distress Among Women during War. J. Soc. Clin. Psychol..

[48] Prati G., Pietrantoni L. (2010). The relation of perceived and received social support to mental health among first responders: A meta-analytic review. J. Community Psychol..

[49] Siriwardhana C., Ali S.S., Roberts B., Stewart R. (2014). A systematic review of resilience and mental health outcomes of conflict-driven adult forced migrants. Confl. Health.

[50] Kien C., Sommer I., Faustmann A., Gibson L., Schneider M., Krczal E., Jank R., Klerings I., Szelag M., Kerschner B. (2019). Prevalence of mental disorders in young refugees and asylum seekers in European Countries: A systematic review. Eur. Child Adolesc. Psychiatry.

[51] Kirmayer L.J., Narasiah L., Munoz M., Rashid M., Ryder A., Guzder J., Hassan G., Rousseau C., Pottie K., for the Canadian Collaboration for Immigrant and Refugee Health (CCIRH) (2010). Common mental health problems in immigrants and refugees: General approach in primary care. Can. Med. Assoc. J..

[52] Hollander A.-C., Bruce D., Burström B., Ekblad S. (2011). Gender-related mental health differences between refugees and non-refugee immigrants—A cross-sectional register-based study. BMC Public Health.

[53] Satinsky E., Fuhr D.C., Woodward A., Sondorp E., Roberts B. (2019). Mental health care utilisation and access among refugees and asylum seekers in Europe: A systematic review. Health Policy.

[54] Connor J., Madhavan S., Mokashi M., Amanuel H., Johnson N.R., Pace L.E., Bartz D. (2020). Health risks and outcomes that disproportionately affect women during the Covid-19 pandemic: A review. Soc. Sci. Med..

[55] Rousseau C., Frounfelker R.L. (2018). Mental health needs and services for migrants: An overview for primary care providers. J. Travel Med..

